# Oncogenic human papillomavirus infection (HPV 16/18) and associated factors among women in East Gojjam Zone, NorthWest Ethiopia 2021

**DOI:** 10.4314/ahs.v24i4.3

**Published:** 2024-12

**Authors:** Mamaru Getinet

**Affiliations:** Department of Biomedical Sciences, School of Medicine, Debre Markos University, Debre Markos, Ethiopia

**Keywords:** OncoE6TM cervical test, HPV, East Gojjam zone, prevalence, associated factors

## Abstract

**Background:**

Human papillomavirus is a common pathogen that infects the skin and mucosal epithelium, is transmitted sexually; causes condylomas or squamous cell carcinomas. Two (16 and 18) of the 150 HPV serotypes are oncogenic types. Studies have been done on the infection by oncogenic human papillomavirus 16/18 and associated factors are found to be very limited in Ethiopia. This study aimed to assess the prevalence of oncogenic human papillomavirus infection (HPV 16/18) and associated factors among women.

**Methods:**

An institutional-based cross-sectional study was conducted among 337 women screened for cervical cancer in two hospitals in East Gojjam Zone from February to April 2021 G.C. Four BSc-qualified nurses who worked in the chosen hospitals collected the data using pretested questionnaire and an HPV test (OncoE6TM Cervical Test) specific to HPV16/18 in cervical swabs. Descriptive analysis was performed to determine the prevalence and a multivariate logistic regression model was used to identify the associated factors of HPV16/18 infection. Finally, statistical significance was declared at P < 0.05.

**Results:**

The prevalence of HPV infection was 14.2% (95% CI: 10.7% -18.1%). The mean age of the respondents was 36.7±9.1 years. Women with the age group of 55-65 years (AOR = 7.91, 95% CI: 1.95-32.09), early initiation of sexual intercourse (AOR = 5.36, 95% CI: 1.58-18.13), history of sexually transmitted infection (STI) (AOR = 3.52, 95% CI: 1.27-9.72), human immunodeficiency virus (HIV) positive status (AOR = 6.8, 95% CI: 1.99-23.54), and number of lifetime sexual partners (AOR = 4.37, 95% CI: 1.15-17.3) were important independent factors associated with the presence of oncogenic HPV infection.

**Conclusion and Recommendation:**

We found a relatively low prevalence of high-risk HPV infection. Age, early initiation of sexual intercourse at less than 18 years, STI of women, being HIV seropositive, and a number of sexual partners were important factors for high-risk HPV infection. Women aged > 46 years, women with early initiation of sex, a history of STI, being HIV positive, and a history of multiple sexual partners should be encouraged to be screened and vaccinated for HPV infection. Wider-ranging studies are also needed in HPV-infected women in association with the cervical lesion.

## Introduction

Human papillomavirus is a common pathogen that infects the skin and mucosal epithelia, transmitted sexually. Two (16 and 18) of the 150 HPV serotypes are oncogenic types. In the anogenital region, HPV infections can cause diseases such as condylomas or squamous cell carcinomas 1,2. Often, our immunity can spontaneously clear off most HPV infections without treatment. However, in an immune deficiency state, the infection progress to the cervical lesion. Finally, the lesion causes cervical cancer, unless it is detected and treated early 3. Several studies showed that 14% of global incidences and 18% of deaths from cervical cancer occur in sub-Saharan African countries and 14%-17% in Ethiopia 4-7. Furthermore, studies in Gurage Ethiopia also showed that the prevalence of HPV infection was 17.3%; which is a relatively high prevalence 8. Contrary to other malignancies of the reproductive system, cervical cancer can be avoided by early detection of a precancerous lesion through screening and treatment of lesions. Any one of the three techniques can be used to identify such lesions; human papillomavirus (HPV) Deoxyribose Nucleic Acid (DNA) test, pap smear, and visual inspection with acetic acid 9,10. Improved screening coverage, vaccination accessibility, early cervical lesion treatment, and identification and monitoring of risk factors are all important in managing the burden of high-risk human papillomavirus infection and associated cervical cancer. The aforementioned strategies significantly reduce cervical cancer-related mortality and morbidity in settings with limited resources 11. In resource-constrained settings, the World Health Organization (WHO) 2013 cervical cancer guideline suggested routine screening for women of reproductive age followed by cryotherapy treatment 12. The likelihood of unvaccinated HPV-negative women developing cervical cancer in the next five to ten years is reduced, according to several studies, indicating that primary HPV testing is a crucial preventive measure, especially for unvaccinated women 13. Ethiopia began implementing preventive measures in September 2010, and the national health strategy included the prevention and control of cervical cancer^14^. Additionally, a pilot program to vaccinate schoolgirls against HPV genotypes 16 and 18 before they have their first sexual experience began in 2018 and included Ethiopia. Cervical lesions and HPV infection are widespread in the community despite the use of such preventive measures. According to my search, until the present study was conducted, the study setting data on the prevalence and risk factors of high-risk HPV infection was not established. The objective of the current study is to determine the prevalence of oncogenic HPV infection, and associated factors among women undergoing cervical cancer screening in hospitals in the East Gojjam zone, northwest Ethiopia. As a result, the study gives information to the relevant authorities for designing intervention plans, such as health promotion and education regarding the prevention and management of HPV infection and associated cervical cancer. The results of this study will serve as the starting point for more research.

## Materials and methods

A facility-based cross-sectional study design was conducted at two hospitals that are located in the East Gojjam Zone from February to April 2021 G.C. The sample size was calculated using a single population proportion formula ( ). Considering the prevalence of HPV at 16% 3 with a 5% margin of error, a 95% confidence level, and a design effect of 1.5; the final sample size was 337. The final sample size was proportionated to selected two hospitals (Debre Markos Comprehensive Referral Hospital (200), and Finote Selam hospital (137)) which provide HPV testing and cervical cancer screening. The study settings were selected from eleven public hospitals (one referral and ten primary hospitals) based on the lottery method. Screening of women of reproductive age for HPV in Ethiopia has been scaled up in all hospitals since 2018.

### Study population

Up until the required sample size was reached, women who had cervical cancer screening in selected hospitals were included in the study. However, to prevent unneeded discomforts associated with the procedure's endocervical swabs, women with verified malignant tumors and pregnant women were excluded from the present study. Data collection procedure and quality assurance Data were collected using a pretested structured questionnaire that was created after reviewing other related studies and being modified following the objectives of this particular study^9^,^11^,^15^-^17^. Data collectors received two days of training on data collection practices. The four supervisors were tasked with assessing the data collection, while the four nurses qualified with BSc who were certified in cervical cancer screening and working in the gynecology ward of the chosen hospitals were assigned to collect the data. The questionnaire was initially written in English before being translated into Amharic. After receiving verbal agreement from the clients in the healthcare institution, the data was collected from them through face-to-face interviews. A trained professional conducted the screening procedure following the HPV infection screening protocol outlined in the guidelines 14. Before the actual data collection, the questionnaire was pretested among 5% of the study population in the Bichena primary hospital to ensure its consistency, completeness, and appropriate modifications were done.

### Data Processing and Analysis

The acquired data were coded, entered, and cleaned using EpiData version 4.6 before being exported into the SPSS version 26 software for analysis after being ensured to be complete. Sociodemographic factors and the prevalence of HPV were summarized using appropriate descriptive analysis techniques such as percentages, summary statistics, and cross-tabulation. Bivariate and multivariate logistic regression were used to assess risk factors associated with the presence of HPV and precancerous cervix lesions. The multivariate logistic regression was used to reanalyze the variables in the bivariate logistic regression that yielded P<0.25. Variables yielding P<0.05 with a 95 % confidence interval were considered statistically significant throughout repeated logistic regression analyses and were associated with the presence of oncogenic HPV infection.

### Ethical approval

The ethical clearance was obtained from the Ethics Committee of the School of Medicine of Debre Markos University (1874/02/2021). Furthermore, permission was obtained from the East Gojjam Zone health department and managers of selected hospitals. According to the Helsinki Declaration, this study was carried out. The informed vrbal consent was obtained from the study participants after being informed about the voluntary basis of participation. All methods used in the study were in line with the regulations and guidelines for the treatment of diseases in hospitals of East Gojjam. The confidentiality of patient information was protected.

### Measurements and Definitions

Oncoprotein E6 HPV 16/18 testing. Women who participated in the study had their endocervical swabs taken by inserting the swab devices into the endocervix and rotating them three times in a counterclockwise direction. According to the manufacturer's instructions, the E6 HPV 16/18 oncoprotein detection lateral flow (LF) strip test (OncoE6TM Cervical Test; Arbor Vita Corporation; Fremont, CA, USA) was used to identify HPV types 16 and 18 in cervical swabs. The test results were also interpreted following the manufacturer's guidelines. Use of a contraceptive technique: Using for more than or equal to one month, such as oral contraceptive pills, an injection, an implant, or intrauterine devices Early sexual initiation: Engage in sexual activity before turning 18 years of ag.

Multiparity: having more than two offspring Multiple partners for sexual activity: Having sexual relations with two or more people at once.

## Results

Sociodemographic Characteristics of participants A total of 337 women aged 25 to 65 years were enrolled in the study with a mean age of 36.7±9.1 years. The response rate of the study participants was 100%. Among the study participants, 153 (45.4%) were in the age group 25 to 35 years. Of the total of respondents, 314 (92.2%) were Orthodox Christian followers followed by protestants. Among the study participants, 328 (97.3%) of them were Amhara and followed by Oromo. Of those respondents, 136 (40.4) and 201 (59.6%) of them resided in rural and urban areas respectively. The majority of 254 (75.4%) were married and 191 (56.7%) were housewives and 135 (40.1%) did not receive a formal education (Table 1).

**Table 1:** Socio-demographic characteristics of the participant women (n=337) aged 25 to 65 years in the East Gojjam Zone, northwest Ethiopia, 2021

Variables and Category	Number	Percentage (%)	Mean ± SD
**Age**			
25-35	153	45.4	**36.7±9.1**
36-45	117	34.7	
46-55	38	11.3	
56-65	29	8.6	
**Religion**			
Orthodox	314	92.2	
Muslim	7	2.1	
Protestant	16	4.7	
**Ethnicity**			
Amhara	328	97.3	
Oromo	9	2.7	
**Residence**			
Urban	201	59.6	
Rural	136	40.4	
**Marital status**			
Married	254	75.4	
Single	23	6.8	
Widowed	21	6.2	
Divorced	39	11.6	
**Education status**			
Diploma and higher	67	19.9	
Secondary (9-12)	51	15.1	
Primary (1-8)	84	24.9	
No formal education	135	40.1	
**Occupation**			
Housewife	191	56.7	
Daily laborer	38	11.3	
Merchants	42	12.5	
Governmental employee	66	19.5	

## Reproductive health characteristics

Among study participants, 229 (68%) of them used contraceptive methods. Of the participants who used contraceptives, 28 (12.3%), 61 (26.6%), 93 (40.6 %), 47 (20.5%) were used intrauterine devices (IUCD), implants, injectables, and pills, respectively. The majority of 183 (54.3%) of the study respondents had an irregular menstrual history in terms of menstrual regularity. 75 respondents reported having experienced postcoital bleeding in the past, whereas the remaining respondents had no such history. The majority of the study participants had given birth to more than three children with a mean number of parity 4.13 + 2.5 children (Table 2).

**Table 2:** Reproductive characteristics of participant women (n=337) aged 25-65 years in the East Gojjam Zone, northwest Ethiopia, 2021

Variables and Category	Number	Percentage (%)	Mean±SD
**Contraceptive use**			
No	108	32	
Yes	229	68	
**Type of contraceptive**			
IUCD	28	12.3	
Implant	61	26.6	
Injectable	93	40.6	
Pills	47	20.5	
**Menstrual history**			
Regular	142	42.1	
Irregular	183	54.3	
No menses	12	3.6	
**Post-coital bleeding**			
No	262	77.7	
Yes	75	22.3	
**Give birth**			
No	64	18.9	
Yes	273	81.1	
**Number of births**			
1-2	33	12	**4.13 ± 2.5**
3-4	213	78.2	
>4	27	9.8	

## Lifestyle and Sexual Behavior

Only 45 (13.4%) of the participants in this study had ever undergone a cervical cancer screening. 7 (2.1%) of the respondents had ever smoked and 22 (6.5%) of them regularly drank alcohol. One hundred and fifty of the respondents had their first sexual intercourse before the age of 18, while the remaining 187 (55.5%) did so when they were at least 18 years old with the mean age at their first sexual intercourse being 17.01 ± 3.5 years. Of the study participants, 265 (78.6%) never used condoms throughout their lifetime. A total of 103 (30.6%) of the study participants had a history of STI, and 113 (33.5%) of them had a husband who had a history of STI. 54 (16%) of the respondents who underwent HIV testing were HIV seropositive. Regarding several sexual encounters, 180 respondents (53.5%) reported having more than two sexual partners, while the rest did not (Table 3).

**Table 3:** Lifestyle and sexual behavior characteristics of the participant women (n = 337) aged 25 to 65 years old in the East Gojjam Zone, northwest Ethiopia, 2021

Variables and Category	Number	Percentage (%)	Mean ± SD
**Previously screened for cervical cancer**			
No	292	86.6	
Yes	45	13.4	
**History of smoking**			
No	330	97.9	
Yes	7	2.1	
**Alcoholic history**			
No	315	93.5	
Yes	22	6.5	
**Age of first sex**			
<18	150	44.5	**17.01 ± 3.5**
≥18	187	55.5	
**Condom use**			
Always	4	1.2	
Sometimes	68	20.2	
Never	265	78.6	
**Ever history of STI**			
No	234	69.4	
Yes	103	30.6	
**Partners' history of STI**			
No	224	66.5	
Yes	113	33.5	
**HIV status**			
Negative	283	84	
Positive	54	16	
**CD4 count in cells per mm^3^**			
<200	18	5.3	**247.2 ± 163.8**
≥200	26	94.7	
**Lifetime sexual partner of women**			
<2	157	46.5	**2.12 ± 1.6**
≥2	180	53.5	

## Prevalence of oncogenic HPV infection

The prevalence of oncogenic HPV infection among women screened for cervical cancer was 14.2% (95% CI: 10.7% - 18.1%).

## Factors Associated with oncogenic HPV infection

Variables yielding P<0.25 after bivariate analysis were deemed confounding factors, and they were reanalyzed in multivariate logistic regression to ascertain their association with oncogenic HPV infection. In multivariate logistic regression analysis, the variables yielding P<0.05 were significantly associated with the presence of oncogenic HPV infection.

## Bivariate logistic regression

The bivariate logistic regression analysis showed that age, rural residence, level of education, contraceptives use, starting sexual intercourse at less than eighteen years, the number of births, abortion, partners' history of STI, history of partner STI, HIV positive serostatus, having two or more lifetime sexual partner significant association with high-risk HPV infection (Table 4).

**Table 4:** Multivariate logistic regression analysis showing factors associated with oncogenic HPV infection among participant women (n=337) aged 25-65 years in the East Gojjam Zone, Northwest Ethiopia, 2021

Variables and Category	HPV infection based on E6 16/18 antigen test				
	Positive n (%)	Negative n (%)	COR (95% CI)	AOR (95% CI)	P value
**Age**					
25-35	11 (7,2)	142 (92.8)	1	1	1
36-45	13 (11.2)	104 (88.9)	0.47 (0.05-0.39)	2.24 (0.65-7.71)	0.19
46-55	14 (36.8)	19 (65.5)	0.23 (0.09-0.61)	4.49 (1.09-18.47)	0.06
56-65	10 (34.5)	289 (85.8)	1.10 (0.40-3.04)	7.91 (1.95-32.09)	0.01*
**Residence**					
Rural	25 (18.4)	111 (81.6)	0.07 (1.74-3.22)	1.33 (0.38-4.61)	0.64
Urban	23 (11.4)	178 (88.6)	1	1	1
**Educational status**					
No formal education	29 (21.5)	106 (78.5)	0.23 (0.07-0.69)	0.34 (0.10-1.19)	0.65
Primary (1-8)	12 (14.3)	72 (85.7)	0.22 (0.06-0.78)	1.43 (0.25-8.04)	0.68
Secondary (9-12)	3 (5.9)	48 (94.1)	0.61 (0.29-1.27)	0.88 (0.17-4.39)	0.87
Diploma and above	4 (6)	63 (94)	1	1	1
**Contraceptive use**					
No	13 (12)	95 (88)	1	1	1
Yes	35 (15.3)	194 (84.7)	0.75 (0.38-1.51)	1.61 (0.58-4.41)	0.35
**Age at first intercourse**					
< 18	41 (24.7)	125 (75.3)	0.14 (0.05-0.34)	5.36 (1.58-18.13)	0.01*
≥ 18	7 (4.1)	164 (95.9)	1	1	1
**Number of births**					
1-2	5 (15.6)	27 (84.4)	1	1	1
3-4	26 (12.1)	188 (87.9)	0.19 (0.06-0.67)	0.31 (0.07-1.31)	0.11
>4	13 (48.1)	14 (51.9)	0.14 (0.06-0.35)	0.92 (0.17-4.93)	0.93
**Abortion**					
No	27 (11.2)	215 (88.8)	1	1	1
Yes	21 (22.1)	72 (77.9)	0.44 (0.23-0.83)	0.51 (1.32-1.89)	0.51
**Women history of STI**					
No	26 (11.1)	208 (88.9)	1	1	1
Yes	22 (21.4)	81 (78.6)	0.46 (0.24-0.85)	3.52 (1.27-9.72)	0.02*
**Partners history of STI**					
No	28 (12.5)	196 (87.5)	1	1	1
Yes	20 (17.7)	93 (82.3)	1.51 (0.86-2.81)	0.43 (0.03-5.64)	0.52
**HIV serostatus**					
Negative	9 (3.2)	274 (96.8)	1	1	1
Positive	39 (72.2)	15 (27.8)	0.01 (0.01-0.03)	6.81 (1.99-23.54)	0.01*
**Number of sexual partners of a woman**					
< 2	5 (3.2)	152 (96.8)	1	1	1
≥ 2	43 (24.3)	134 (75.7)	9.55 (3.75-25.34)	4.37 (1.15-17.3)	0.03*

**Legend:** (*) --Variables that were significantly associated with oncogenic HPV infection

(1) …. Reference category

## Multivariate Logistic Regression

As clearly stated in table 4, all variables in the table have a P value of less than 0.25 in bivariate logistic regression and are reanalyzed in multivariate logistic regression analysis. Controlling for the effect of confounders age group 56-65 years, starting sexual intercourse at less than 18 years, STI of women, being HIV seropositive, and having multiple sexual partners were found to be significantly associated with the presence of oncogenic HPV infection. Women in the age group from 56 to 65 years were 7.9 times more likely to have high-risk HPV infection compared to the age group of 25 to 35 years (AOR = 7.91, 95% CI: 1.95-32.09). Regarding early initiation of sexual intercourse, those women stats sex before age less than 18 were 5.3 times riskier to develop oncogenic HPV infection than their counterparts. Women who had a history of STI were 3.52 times more likely to have a high-risk HPV infection as compared to those who had no history of STI (AOR = 3.52, 95% CI: 1.27-9.72). Women who had HIV-positive serostatus were 6.8 times more likely to have a high-risk HPV infection compared to HIV negatives (AOR = 6.8, 95% CI: 1.99-23.54). Women who had two and more lifetime sexual partners were 4.37 times more likely to develop high-risk HPV infection compared to those who had less than two sexual partners (AOR = 4.37, 95% CI: 1.15-17.3).

## Discussion

We assessed oncogenic HPV infections, and associated factors among women 25-65 years of age. The prevalence of high-risk HPV infection was 14.2% which was lower than the study conducted by Gebremariam T et.al 16% 3, in other East African countries, Mozambique (40.3%) [18] and Kenya (41.4%) 7 and higher than a study done in Sudan (3.2%). This disparity could be the result of the abilities of the test provider, the differing length of the study time, and the study's exclusion of pregnant women with cervical cancer and carrying a child. We employed an antigen detection method, whereas others used a molecular detection method to detect many HPV types. In the world, HPV types 16 and 18 are the most prevalent and are to blame for the majority of anogenital HPV-related malignancies in women^19^.

The current study also assessed factors associated with the presence of oncogenic HPV infection among the study participant and the findings revealed that respondents who had an age group 56-65 years, started sexual intercourse at less than 18 years, STI in women, being HIV seropositive, and having multiple sexual partners were predictors of oncogenic HPV infection. According to the findings of the current study, women between the ages of 56 and 65 were 7.9 times more likely to have high-risk HPV infections than women between the ages of 25 and 35. This finding is consistent with other studies findings^20^-^24^. This might be due to immune deficiency that is predominant in advanced age and cause multiple infections. According to the current study, the average age of the started first sexual intercourse of participants was 17.01, which was closer to the average age of their first marriage, which was 16.8 years. This implies that women generally start having sex after getting married for the first time. A little more than 150 respondents (44.5%) had their first sexual experience before turning 18 years old. Our finding showed women's start sexual intercourse before the age of 18 were 5.36 times more likely to have the chronic infection of oncogenic HPV infection; that is consistent to s other studies done in different settings^25^-^29^. This might be a result of the extended duration of HPV virus exposure and the slow onset of precancerous cervical abnormalities^30^,^31^.

Co-infections of HPV and other STDs may be the reason for this connection. The present study similar to the studies from Zambia 32 and Rwanda 33 revealed a high prevalence of HPV (72.2%) among HIV-positive women than among women without HIV (3.2%). Furthermore, women who had HIV-positive serostatus were 6.8 times more likely to have oncogenic HPV infection compared to HIV negatives. Our finding is strongly supported by the studies done in Tanzania^34^, Uganda^35^, and southern Ethiopia 25. This association might be due to HIV infection being an immune suppressor disease that increases the probability of concurrent HPV infection 36.

STIs are a sign of having unsafe sex, which is how genital human HPV is primarily transmitted. Concurrent to this, we found a slightly higher prevalence of HPV infections among women who had a history of STIs (12.5%) than those who had not (17.7%). Moreover, having multiple sexual partners had 11.5 times higher odds of oncogenic HPV infections. These findings are consistent with the findings of studies conducted by different authors in different countries [23, 25, 29, 37-40]. The possible explanation is due to the incidence of having multiple sexual partners increasing the likelihood of acquiring HPV infection, which in turn, causes cervical cancer.

## Conclusions

The present study identified a relatively low prevalence of oncogenic HPV 16/18 and cervical lesions and pre-cancerous cervical lesion. According to the current study, testing for HPV increases the early identification of highrisk women for effective cervical cancer screening programs. This study revealed that the age group of 55-65 years, early initiation of history of STI, being HIV positive, and a number of sexual partners; all are independently associated factors for the presence of oncogenic HPV infection. Therefore, use outreach cervical cancer screening programs to encourage more women to participate in screening and vaccination. Moreover, it is advised that all women who are older than 55 and have a history of STIs, HIV, and several sexual partners get screened and treat for oncogenic HPV infection. Strong policies and guidelines must also be developed for the prevention and control of oncogenic HPV infection and associated cervical cancer. A wider range of studies is also needed to understand VIA positive in HPV-infected women.

## Data Availability

Data used to support the finding of this study are available from the corresponding author upon request.

## Abbreviations

CD4: Cell differentiation 4
EDHS: Ethiopian Demographic Health Survey
HPV: Human Papilloma Virus
HIV: Human Immune Virus
IUCD: Intrauterine Contraceptive Device
SPSS: Statistical Package for Social Science
STI: Sexually transmitted infection
VIA: Visual Inspection with Acetic Acid
